# Recurrent myocarditis masquerading as acute myocardial infarction: unmasking Behçet’s disease

**DOI:** 10.1093/omcr/omag186

**Published:** 2026-10-04

**Authors:** Mohamed Atta, Fadel Alqatati, Mohammed Alkhanafsa, Kholoud Riyad Jaber, Mhd Baraa Habib

**Affiliations:** Department of Cardiology, Heart Hospital, Hamad Medical Corporation, Doha 3050, Qatar; Department of Internal Medicine, Hamad Medical Corporation, Doha 3050, Qatar; Department of Internal Medicine, Hamad Medical Corporation, Doha 3050, Qatar; Faculty of Medicine, Alexandria University, Alexandria 21526, Egypt; Department of Cardiology, Heart Hospital, Hamad Medical Corporation, Doha 3050, Qatar

**Keywords:** myocarditis, cardiac magnetic resonance, troponin, Behçet’s disease, colchicine

## Abstract

Myocarditis can closely mimic acute myocardial infarction (AMI), particularly in young patients without atherosclerotic risk factors. We report a 27-year-old man with recurrent severe chest pai7n, transient ST-segment elevations, and markedly elevated high-sensitivity troponin-T, in whom coronary angiography was repeatedly normal. Serial cardiac magnetic resonance and 18F-fluorodeoxyglucose positron-emission tomography revealed a migratory pattern of myocardial inflammation, with new lesions emerging as earlier ones resolved, fulfilling criteria for recurrent myocarditis. Recurrent oral ulcers, folliculitis, arthralgias, and pathergy-like skin reactions raised suspicion for Behçet’s disease. Treatment with vasodilators, colchicine, corticosteroids, and azathioprine produced sustained clinical and biochemical remission. This case highlights the importance of considering myocarditis in young patients with recurrent angina-like symptoms and normal coronaries, the value of serial multimodal imaging in detecting migratory inflammation, and the recognition of Behçet’s disease as a rare underlying cause of recurrent myocarditis.

## Introduction

Myocarditis is an inflammatory myocardial disease whose presentation ranges from subclinical illness to fulminant heart failure and sudden cardiac death, particularly in young adults [1]. Among young patients with myocardial infarction and non-obstructive coronary arteries (MINOCA), myocarditis accounts for approximately one-third of cases, frequently producing chest pain, high-sensitivity troponin-T (hs-TnT) elevation, and transient ST-segment changes that mimic acute myocardial infarction (AMI) despite angiographically normal coronaries [2].

Recurrent myocarditis, defined by discrete flares separated by symptom-free intervals, is increasingly recognised and may reflect cytokine-mediated coronary vasospasm, microvascular dysfunction, or systemic vasculitis such as Behçet’s disease [1, 3]. Cardiac magnetic resonance (CMR), interpreted with the Lake Louise criteria, is the diagnostic cornerstone, while 18F-fluorodeoxyglucose positron-emission tomography (FDG-PET) detects metabolically active inflammation [4, 5]. We report a young man with three infarct-like flares of recurrent migratory myocarditis whose presentation ultimately unmasked Behçet’s disease.

## Case report

A 27-year-old previously healthy Egyptian man, with no cardiovascular risk factors, presented with three days of severe left-sided chest pain radiating to the neck and associated diaphoresis, without fever or viral prodrome. He reported occasional oral aphthae and folliculitis. Family history included premature myocardial infarction in an uncle at age 31 and paternal ankylosing spondylitis. He was haemodynamically stable (blood pressure 142/96 mmHg, heart rate 83 bpm); cardiopulmonary examination was unremarkable.

The initial electrocardiogram (ECG) showed normal sinus rhythm (Fig. 1a). hs-TnT was markedly elevated at 354 ng/L (normal < 15), with C-reactive protein 17 mg/L and a normal erythrocyte sedimentation rate; enterovirus and parvovirus B19 polymerase chain reaction were negative. On serial measurement, C-reactive protein remained persistently around the upper limit of normal (peak 17 mg/L; reference range 0–15 mg/L) and did not correlate with symptom severity, troponin peaks, clinical improvement at discharge, or relapse. Urgent coronary angiography demonstrated normal coronaries. Echocardiography showed preserved left ventricular ejection fraction (LVEF ≈55%), but CMR the next day revealed mildly reduced LVEF (≈43%), T2 myocardial oedema, and subepicardial late gadolinium enhancement (LGE) in the anterior and anterolateral walls, consistent with acute myocarditis (Fig. 2).

**Figure 1:**
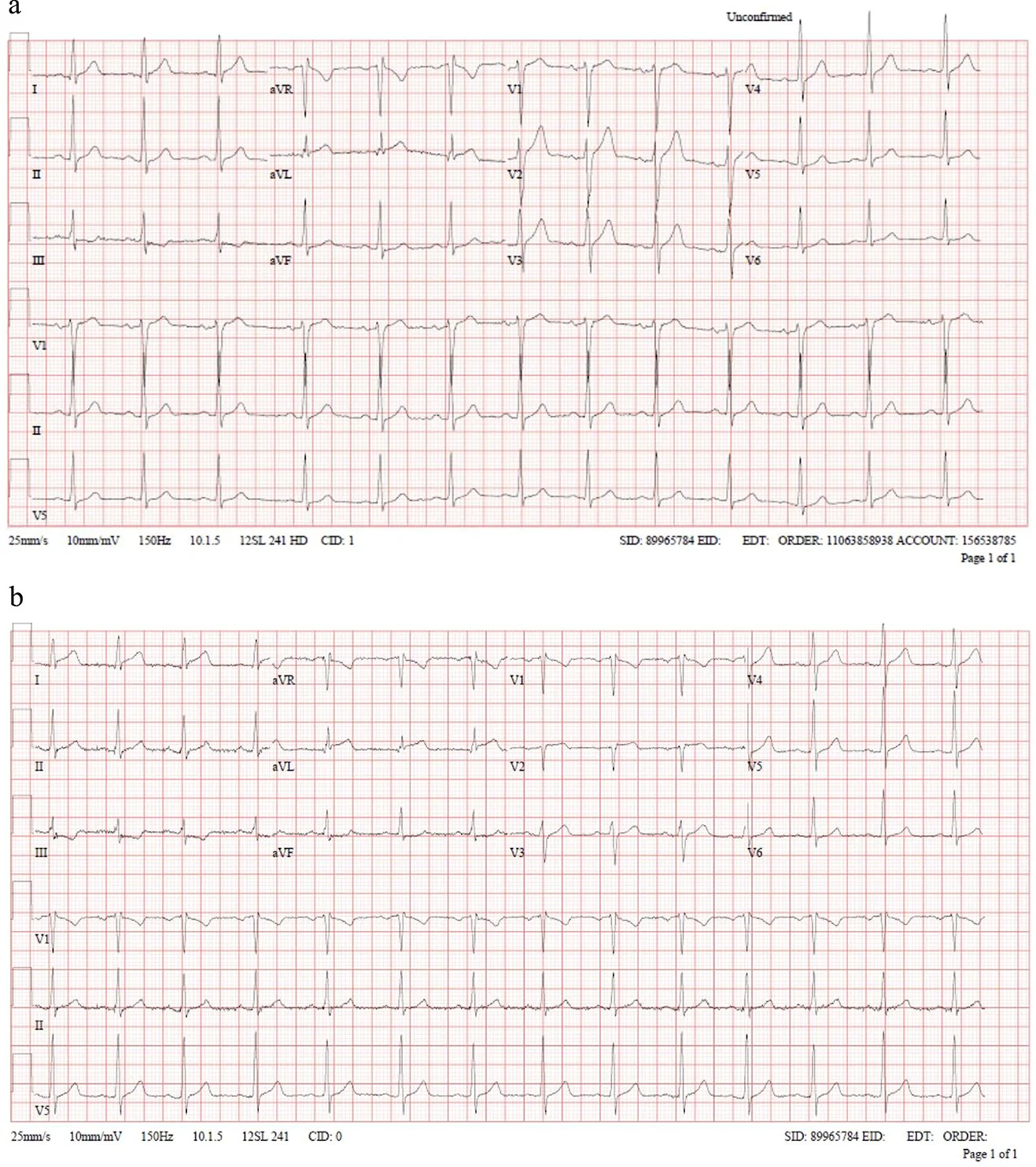
(a) Electrocardiogram on first presentation: Normal sinus rhythm without ST-segment changes. (b) Electrocardiogram on day 3: ST-segment elevation in the lateral leads.

**Figure 2:**
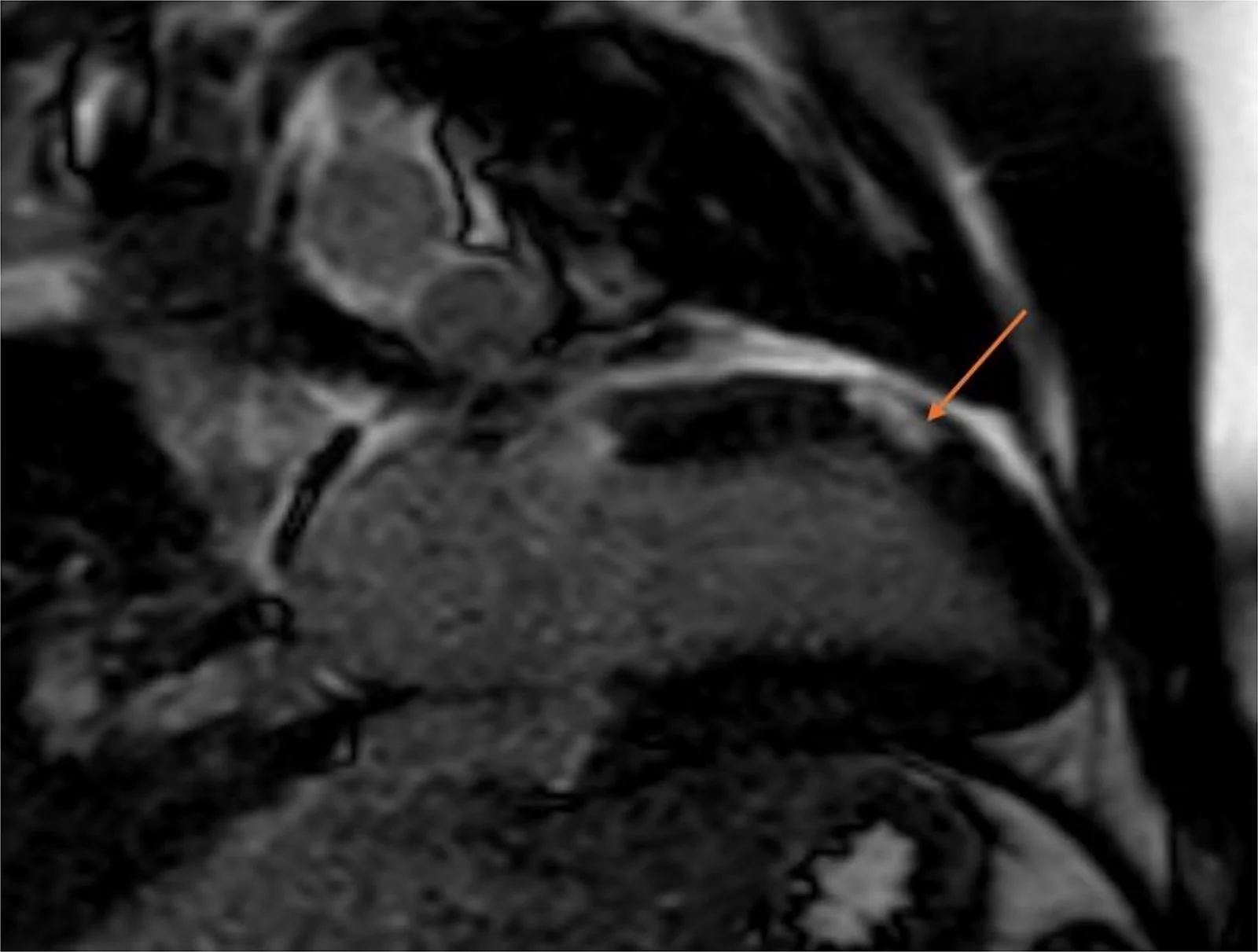
Cardiac magnetic resonance, (day 2), two-chamber phase-sensitive inversion-recovery sequence, showing subepicardial late gadolinium enhancement in the anterior and anterolateral segments (arrow) with myocardial oedema on T2-weighted imaging.

On day 3, recurrent chest pain occurred with lateral ST-segment elevation (Fig. 1b) and hs-TnT rising to 499 ng/L. Intravenous isosorbide dinitrate, oral diltiazem, and analgesia were administered; symptoms settled and hs-TnT fell to 289 ng/L by day 5, when he was discharged.

Eighteen days later he re-presented; hs-TnT was 866 ng/L, peaking at 1006 ng/L. Computed tomography coronary angiography excluded atherosclerosis. Repeat CMR (day 21) showed resolution of the anterior lesions but new LGE in the mid-septal, apical-inferior, and lateral segments (Fig. 3). Colchicine 0.5 mg twice daily produced symptom relief and a fall in hs-TnT to 96 ng/L.

**Figure 3:**
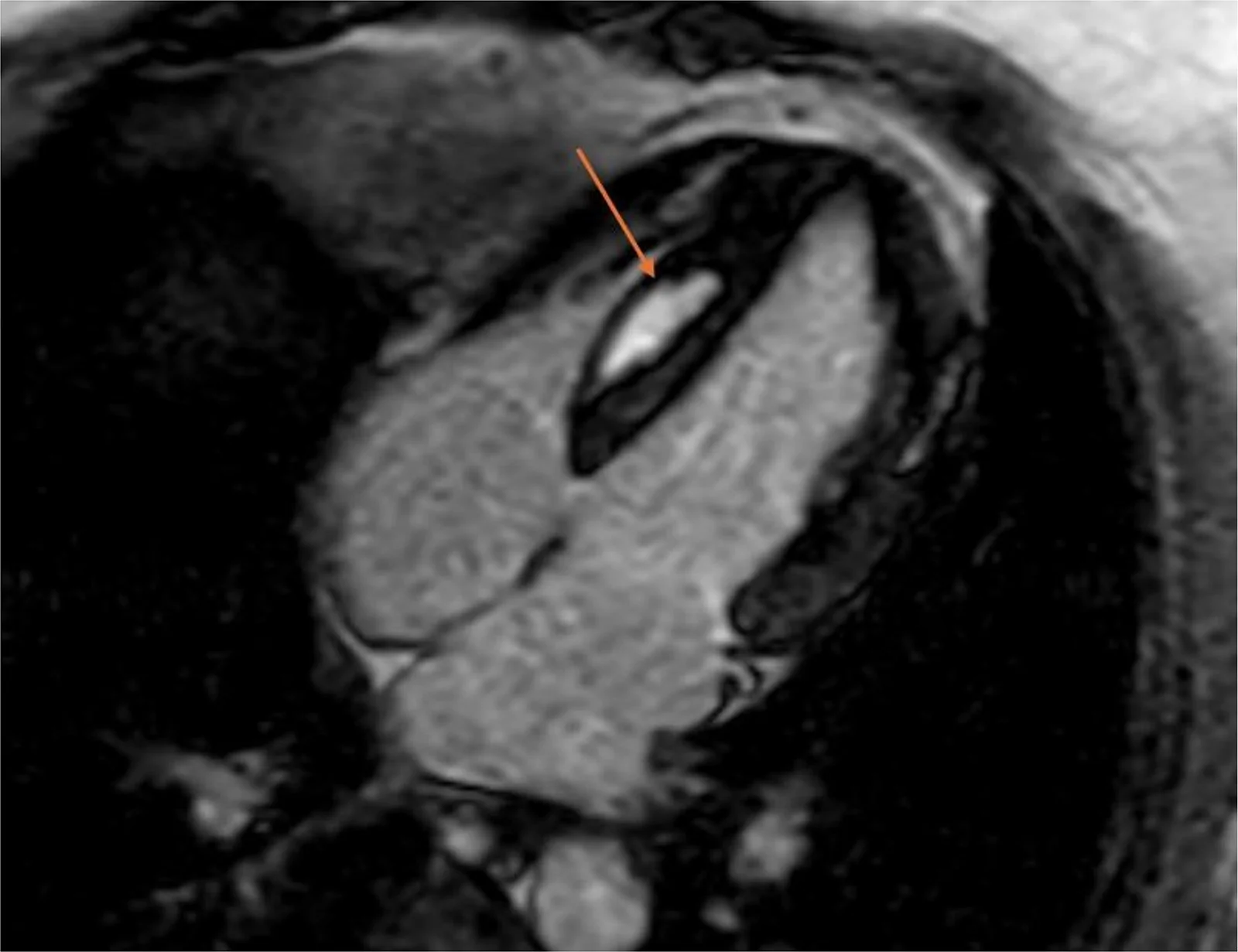
Cardiac magnetic resonance, (day 21), four-chamber phase-sensitive inversion-recovery sequence, showing new late gadolinium enhancement in the mid-septal, apical-inferior, and lateral segments (arrow); previous anterior-wall lesions had resolved.

A third recurrence on day 33 (hs-TnT 633 ng/L) prompted a third CMR (day 37) showing partial regression with new inferolateral and basal-inferior oedema and LGE. Endomyocardial biopsy revealed interstitial oedema with CD3-positive lymphocytes and CD68-positive macrophages, without granulomas or giant cells; cytomegalovirus immunohistochemistry was negative, with clinically insignificant low-level human herpesvirus-7 DNA. FDG-PET (day 40) showed increased uptake in the inferolateral, septal, and apical myocardium (Fig. 4). Antinuclear, antineutrophil cytoplasmic, and anti-double-stranded DNA antibodies were negative. The antistreptolysin-O titre was below the laboratory’s positive threshold (214 IU/mL; positive > 408 IU/mL, borderline 214–407 IU/mL), and blood cultures, a respiratory viral panel, and procalcitonin were all negative. Transthoracic echocardiography, repeated at each admission, consistently showed no significant valvular disease. Recurrent oral ulcers, folliculitis, arthralgias, and delayed cutaneous reactions at cannulation sites (pathergy-like) raised suspicion for Behçet’s disease.

**Figure 4:**
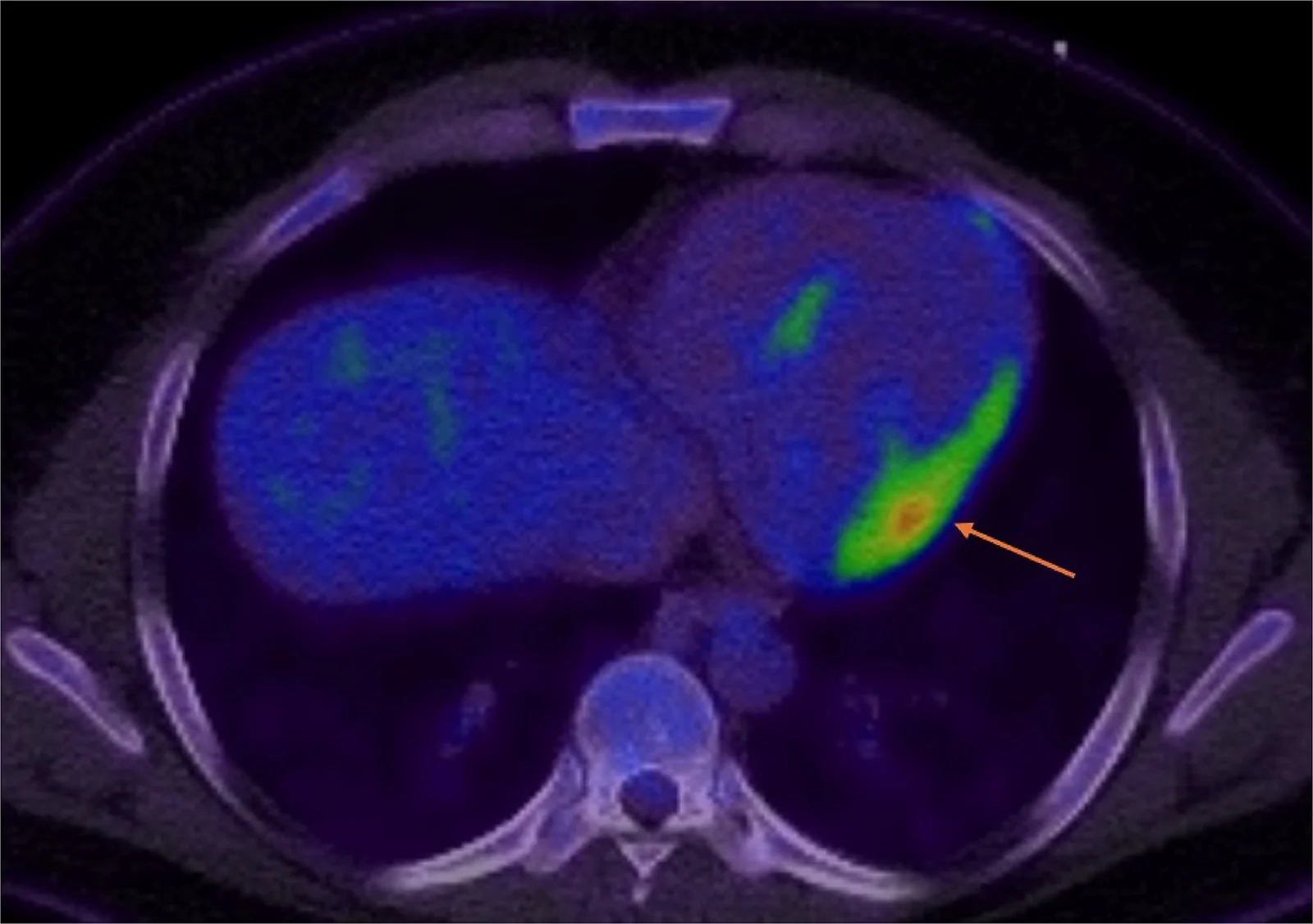
18F-fluorodeoxyglucose positron-emission tomography (day 40) showing intense uptake in the inferolateral wall and the septal and apical myocardium, indicating active inflammation.

After rheumatology review, treatment was escalated to pulsed methylprednisolone (1 g daily for three days) followed by an oral prednisolone taper and intravenous immunoglobulin (2 g/kg over four days) for induction; azathioprine was introduced and uptitrated to 200 mg/day over three months. hs-TnT fell to 58 ng/L by day 50 and normalised to 13 ng/L by day 85 (Table 1, Fig. 5). Corticosteroids were tapered over six months. Surveillance CMR at 13 months showed improved LVEF (≈51%) with resolution of oedema. At 18 months he remained asymptomatic on azathioprine and bisoprolol, off corticosteroids, without further biomarker elevation.

**Table 1:** Serial high-sensitivity troponin-T values with corresponding clinical events.

Day	hs-TnT (ng/L)	Clinical presentation/event
0	354	Initial ED visit: chest pain, normal ECG; myocarditis diagnosed
3	499	1st recurrence: chest pain with lateral ST-elevation
5	289	Improvement; discharged with troponin trending down
18	866	2nd ED visit: recurrent chest pain, raised troponin
19	**1006**	Peak hs-TnT during 2nd flare
21	730	CMR: new LGE; previous anterior lesions resolved
32	96	Improvement on colchicine
33	633	3rd recurrence: chest pain, raised troponin
37	510	CMR: new inferolateral/basal-inferior inflammation
40	430	FDG-PET: active myocardial inflammation
50	58	Improvement on corticosteroids + azathioprine
85	**13**	Follow-up: asymptomatic, normalised hs-TnT, LVEF ~ 51%

hs-TnT, high-sensitivity troponin-T (upper limit of normal < 15 ng/L); ECG, electrocardiogram; CMR, cardiac magnetic resonance; FDG-PET, 18F-fluorodeoxyglucose positron-emission tomography; LGE, late gadolinium enhancement; LVEF, left ventricular ejection fraction.

**Figure 5:**
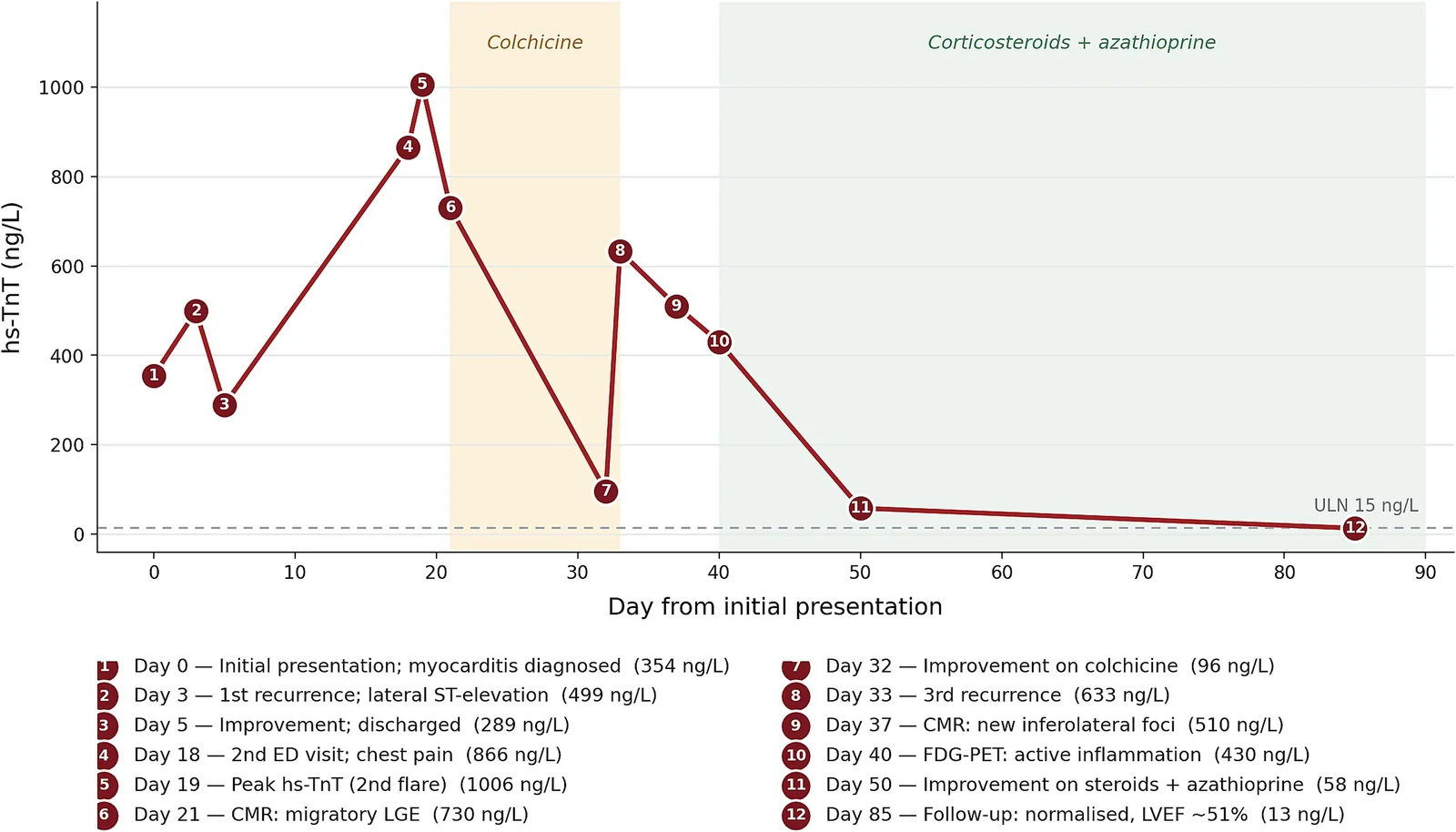
Clinical event timeline summarising symptom recurrences, serial high-sensitivity troponin-T values, imaging, and treatment.

## Discussion

This case illustrates the diagnostic challenge of myocarditis presenting with recurrent chest pain, marked hs-TnT elevation, and transient ST-segment elevation mimicking AMI in a young adult without atherosclerotic risk factors. Registry data indicate myocarditis underlies roughly one-third of MINOCA presentations in this group [2]; it therefore warrants active consideration once obstructive coronary disease is excluded.

Multimodal imaging was decisive. Serial CMR demonstrated a migratory pattern, with new inflammatory foci emerging as earlier lesions resolved, while FDG-PET confirmed metabolically active inflammation, together establishing recurrent myocarditis [4, 5]. Although the three flares occurred over a relatively short interval, they were separated by symptomatic and biochemical improvement and, on serial CMR, involved anatomically distinct territories as earlier lesions resolved; the process was therefore both recurrent and migratory rather than a single evolving lesion. C-reactive protein, by contrast, remained near-normal and dissociated from disease activity throughout, underscoring that a normal inflammatory marker does not exclude active myocardial inflammation, with serial imaging and troponin proving more reliable indicators of relapse. Near-complete regression of LGE at 13 months suggested a favourable prognosis.

Several mechanisms may explain infarct-like recurrences. Cytokine-mediated coronary vasospasm, described in parvovirus B19 myocarditis, can cause angina, transient ST-segment elevation, and biomarker release; the rapid response to nitrates and a calcium-channel blocker supports a vasomotor component [3]. Microvascular dysfunction from interstitial oedema may further impair perfusion.

Alternative aetiologies were systematically excluded. Viral infection is the most commonly presumed cause of myocarditis, with parvovirus B19, human herpesvirus-6, and enteroviruses among the agents most frequently implicated in Europe and North America [1]. In this patient, enterovirus and parvovirus B19 polymerase chain reaction were negative and cytomegalovirus immunohistochemistry was negative; only low-level human herpesvirus-7 DNA was detected, a finding of uncertain significance, as cardiotropic herpesviruses can persist in the myocardium of asymptomatic individuals and are frequently found in controls. The absence of a viral prodrome, the relapsing migratory course, and the accompanying mucocutaneous features argued against a simple post-viral aetiology. Acute rheumatic fever was likewise considered and excluded: the antistreptolysin-O titre was below the positive threshold, blood cultures and procalcitonin were negative, and transthoracic echocardiography at each admission showed no valvular disease or features of endocarditis.

The systemic phenotype, comprising recurrent oral ulcers, folliculitis, arthralgias, and pathergy-like reactions, pointed to Behçet’s disease. Cardiac involvement in Behçet’s disease is rare but varied, encompassing myocarditis, endomyocardial fibrosis, valvular disease, and intracardiac thrombosis, with small-to-medium-vessel vasculitis and vasospasm as putative mechanisms [6]. Although the patient scored only three points on the International Criteria for Behçet’s Disease, below the diagnostic threshold of four (Table 2) [7], the overall clinical constellation with biopsy-proven lymphohistiocytic infiltration justified immunomodulatory therapy.

**Table 2:** International criteria for Behçet’s disease.

Sign/symptom	Points	Patient (points scored)
Ocular lesions	2	–
Genital aphthosis	2	–
Oral aphthosis	2	**Present (2)**
Skin lesions	1	**Present (1)**
Neurological manifestations	1	–
Vascular manifestations	1	–
Positive pathergy test^a^	1	Not formally tested
**Total score**	**≥4 = diagnostic**	**3**

A score of ≥4 points is diagnostic. The patient scored 3 points (oral aphthosis and skin lesions).

^a^The pathergy test is optional; where performed, 1 additional point may be added for a positive result.

Management of Behçet-associated recurrent myocarditis is not standardised. High-dose corticosteroids are first-line for acute flares, with steroid-sparing agents such as azathioprine for maintenance, consistent with EULAR recommendations [8]. Colchicine is well established for acute and recurrent pericarditis [9]; its introduction here coincided with a sustained biochemical and clinical response. These findings parallel Moura *et al*., who reported a 26-year-old with Behçet-related recurrent myocarditis that resolved on colchicine and azathioprine [10].

In conclusion, clinicians should consider recurrent migratory myocarditis in young patients with recurrent angina-like symptoms, transient ST-segment elevation, raised biomarkers, and normal coronaries. Serial CMR with FDG-PET is invaluable for characterising dynamic inflammation and monitoring treatment response, and evaluation for systemic vasculitis such as Behçet’s disease is essential, since timely immunomodulation can prevent progression to irreversible cardiomyopathy.

## Consent

Written informed consent for publication of this case report and the accompanying images was obtained from the patient, in line with COPE guidance.

## Guarantor

Mohamed Atta is the guarantor of this work.

## Data Availability

Data sharing is not applicable to this article, as no datasets were generated or analysed during the current study.
